# Substance Abuse: A growing issue in adolescent population

**DOI:** 10.1192/j.eurpsy.2025.1106

**Published:** 2025-08-26

**Authors:** M. N. Bogdanovic, S. Brozan, A. Kaštelan, D. Petrić

**Affiliations:** 1 CHC Rijeka, Rijeka, Croatia

## Abstract

**Introduction:**

Substance abuse among adolescents has risen significantly in recent years, with serious implications for mental health and development. Factors such as peer pressure, socio-economic stress, and mental health issues contribute to this trend. Early substance use is linked to long-term risks, including substance use disorders and co-occurring psychiatric conditions. This study examines data from CHC Rijeka, highlighting trends in adolescent substance use over recent years, emphasizing the need for targeted prevention and treatment strategies.

**Objectives:**

This study aims to analyze the number of adolescent patients having issues with drug abuse, who were hospitalized at the Adolescent Psychiatry ward in order to identify trends, assess associated risk factors.

**Methods:**

We compared the numbers of hospitalized patients who were dignosed with substance abuse, as well as the percentages of those patients per year, from 2019 until 2024.

**Results:**

From 2019 to 2024, the number of adolescents hospitalized for substance abuse at the Adolescent Psychiatry ward increased. In 2019, substance abuse was diagnosed in approximately 20% of all adolescent admissions, whereas by 2024, this figure had risen to 28%. This rise in the percentage of substance abuse diagnoses, relative to the total number of admissions, indicates a growing prevalence of substance use disorders among hospitalized adolescents. This trend mirrors broader national patterns of increasing adolescent substance use and underscores the urgent need for targeted prevention and intervention strategies, both in clinical settings and within the community.

**Conclusions:**

The rising percentage of adolescents hospitalized for substance abuse highlights a critical public health issue. To address this, efforts should focus on early intervention, increasing access to mental health and substance use services, and implementing school-based prevention programs. Additionally, enhancing community support networks and reducing the stigma around substance abuse treatment are key to preventing further escalation of this growing trend.

**Disclosure of Interest:**

None Declared

